# Risk factors for hemorrhagic cystitis in children undergoing hematopoietic stem cell transplantation: a systematic review and meta-analysis

**DOI:** 10.1186/s12887-024-04815-x

**Published:** 2024-05-14

**Authors:** Senlin Zhang, Minyuan Liu, Qingwei Wang, Shuran Wang, Xin Liu, Bohan Li, Jie Li, Junjie Fan, Shaoyan Hu

**Affiliations:** 1 Department of Hematology and Oncology, Children’s Hospital of Soochow University, No. 92, Zhongnan Street, Suzhou, 215000 China; 2 Jiangsu Pediatric Hematology &Oncology, Suzhou, 215000 China

**Keywords:** Hemorrhagic cystitis, Hematopoietic stem cell transplantation, Children, Risk factors

## Abstract

**Background:**

The risk factors for hemorrhagic cystitis (HC) in children undergoing hematopoietic stem cell transplantation (HSCT) are unclear. Therefore, we conducted this systematic review and meta-analysis to investigate the risk factors for HC in children undergoing HSCT.

**Methods:**

We performed this meta-analysis by retrieving studies from PubMed, EMBASE, and the Cochrane Library up to October 10, 2023, and analyzing those that met the inclusion criteria. I^2^ statistics were used to evaluate heterogeneity.

**Results:**

Twelve studies, including 2,764 patients, were analyzed. Male sex (odds ratio [OR] = 1.52; 95% confidence interval [CI], 1.16–2.00; *p* = 0.003, I^2^ = 0%), allogeneic donor (OR = 5.28; 95% CI, 2.60–10.74; *p* < 0.00001, I^2^ = 0%), human leukocyte antigen (HLA) mismatched donor (OR = 1.86; 95% CI, 1.00–3.44; *p* = 0.05, I^2^ = 31%), unrelated donor (OR = 1.58; 95% CI, 1.10–2.28; *p* = 0.01, I^2^ = 1%), myeloablative conditioning (MAC) (OR = 3.17; 95% CI, 1.26–7.97; *p* = 0.01, I^2^ = 0%), busulfan (OR = 2.18; 95% CI, 1.33–3.58; *p* = 0.002, I^2^ = 0%) or anti-thymoglobulin (OR = 1.65; 95% CI, 1.07–2.54; *p* = 0.02, I^2^ = 16%) use, and cytomegalovirus (CMV) reactivation (OR = 2.64; 95% CI, 1.44–4.82; *p* = 0.002, I^2^ = 0%) were risk factors for HC in children undergoing HSCT.

**Conclusions:**

Male sex, allogeneic donor, HLA-mismatched, unrelated donor, MAC, use of busulfan or anti-thymoglobulin, and CMV reactivation are risk factors for HC in children undergoing HSCT.

**Supplementary Information:**

The online version contains supplementary material available at 10.1186/s12887-024-04815-x.

## Background

Hematopoietic stem cell transplantation (HSCT) is an effective therapeutic approach for various hematologic malignant and nonmalignant diseases [1]. However, the range of complications that arise following HSCT remains a challenge.

Hemorrhagic cystitis (HC) is a frequent complication in patients undergoing HSCT, significantly diminishing their quality of life and extending the length of their hospital stay [2]. Moreover, severe HC is associated with higher mortality [3]. HC is categorized into two groups based on its onset time: early-onset (within 72 h) and late-onset (more than 72 h after conditioning) [4]. The reported incidence rates of HC in pediatric HSCT recipients vary between 3% and 27% [5, 6]. The clinical presentation of HC varies. Mild cases may manifest only as microscopic hematuria, whereas severe cases can manifest as life-threatening complications characterized by continuous bleeding and urinary tract obstruction [7]. The risk factors for HC include age, receipt of cells from an unrelated donor, and the occurrence of acute graft-versus-host disease (GVHD) [8, 9]. However, the conclusions have been inconsistent [10–12], and the risk factors for HC in children undergoing HSCT remain elusive. Therefore, we conducted this systematic review and meta-analysis to further investigate the risk factors for HC, with the aim of providing clinical evidence for early identification and prevention and improving the quality of life of pediatric patients with HC.

## Methods

This study was conducted in accordance with the recommendations of the Preferred Reporting Items for Systematic Reviews and Meta-Analyses (PRISMA) guidelines [13].

### Literature search strategy

The PubMed, EMBASE, and the Cochrane Library databases were searched up to October 10, 2023 by two independent reviewers (Zhang and Wang) for relevant references. Relevant studies were identified using MeSH terms and keywords for cystitis, HSCT, child, pediatric, and adolescent. A detailed literature search process in PubMed is shown in the Supplemental File. The references cited by the included articles were also retrieved to identify other potentially eligible studies. A third reviewer (Fan) was consulted to resolve any disagreements in this process.

### Inclusion and exclusion criteria

The studies were included if they met all the following inclusion criteria: (1) retrospective or prospective original studies published in English; (2) performed among pediatric and young patients aged 21 years or younger; (3) clearly included an HC group and a no HC group; (4) provided sufficient information on the risk factors for HC. Studies were excluded if they met any of the following exclusion criteria: (1) included fewer than five patients in any group; (2) investigated only one category of HC; (3) did not report at least one risk factor for HC; or (4) were reviews, letters, conferences, comments, or case reports.

### Data extraction and quality assessment

A standardized Excel table was used by two investigators to collect the baseline data of the included studies. The data included the first author, publication year, geographic region, study accrual period, study design, sample size of each group, definition of HC, and risk factors reported in the included studies. Two reviewers independently applied the Newcastle–Ottawa scale (NOS) to assess the quality of the included studies. The quality of the study was rated high if the final NOS score was > 7. Any discrepancies in these two processes were resolved through discussion with a third reviewer.

### Statistical analysis

All statistical analyses included in this meta-analysis were performed using Cochrane Review Manager software (RevMan version 5.4). The odds ratios (ORs) and 95% confidence intervals (CIs) were calculated to estimate the roles of all potential risk factors in the development of HC. The heterogeneity of each result was tested using the Cochrane Q test and I^2^ statistics. An I^2^ value < 50% and a P value > 0.1 indicated non-significant heterogeneity, and a fixed-effects model was used. In contrast, an I^2^ value > 50% or a P value < 0.1 indicated significant heterogeneity, and a random-effects model was used. In addition, the publication bias of the included studies was evaluated using funnel plots of each risk factor. An asymmetrical shape of the funnel plot indicates publication bias.

## Results

### Study selection and characteristics

**Fig. 1:**
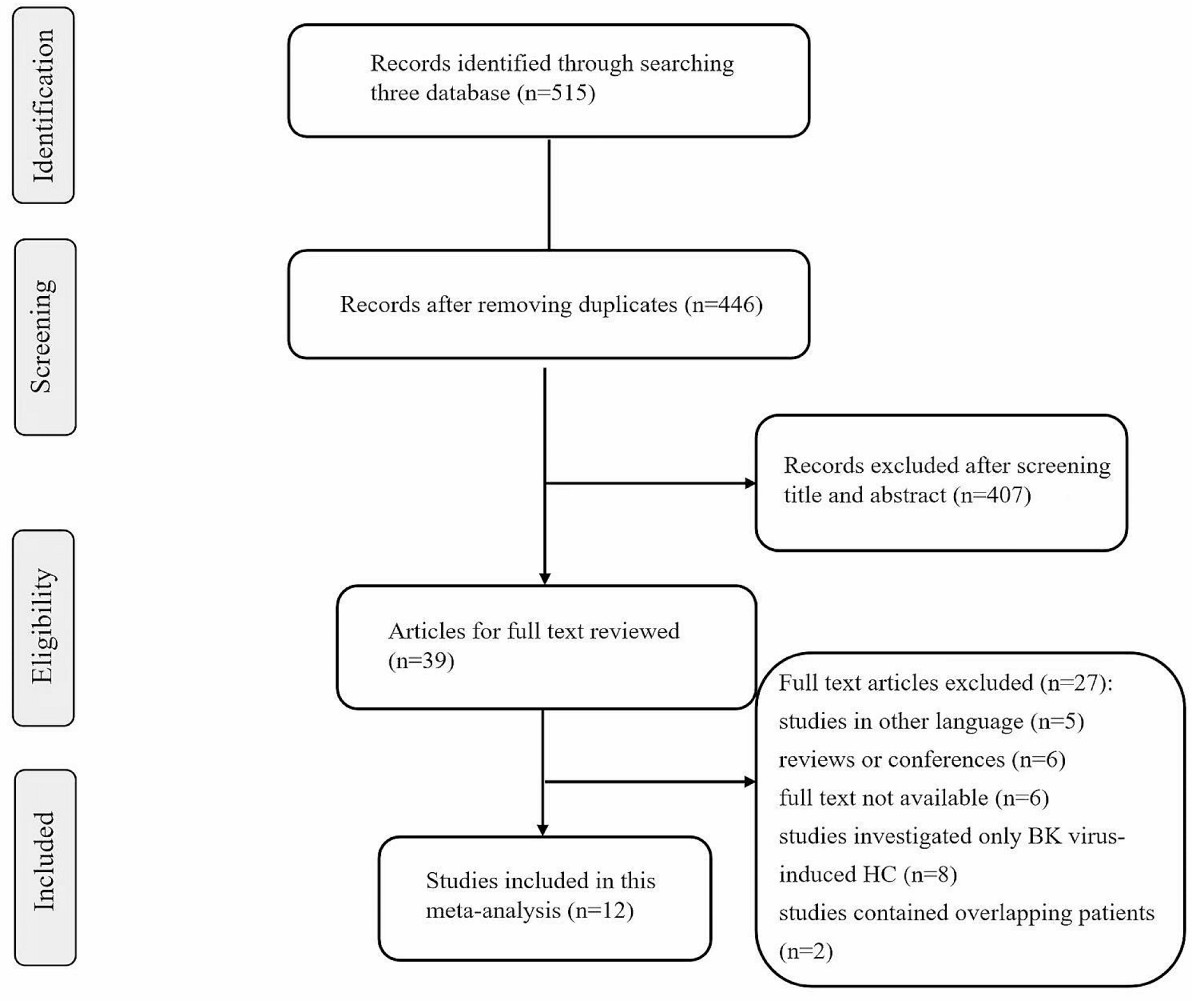
Flow chart of study selection

A total of 515 references were included after the initial search using our search terms (77 in PubMed, 419 in EMBASE, and 19 in the Cochrane Library). After removing 69 duplicate references, the remaining 446 references were further screened by reading the title and abstract. A total of 39 studies were eligible for full-text review, and 12 were included in this meta-analysis [5, 6, 9–11, 14–20]. One study [21] was excluded because it contained data duplicated from another study included in our meta-analysis [15]; therefore, we included the newest data in the analysis. The selection flowchart of the included studies is shown in Fig. 1. Of the 12 included studies, nine were retrospective, and three were prospective, with the publication year ranging from 1998 to 2023. A total of 2,760 pediatric HSCT recipients were included in this meta-analysis, with 290 patients in the HC group and 2,470 patients in the no HC group. Table 1 presents the baseline characteristics and NOS scores for each study. The NOS scores ranged from 6 to 9. Therefore, the quality of the included studies was rated as moderate to high (Table S1). The funnel plots of all risk factors were nearly symmetrical, indicating no publication bias (Figure S1). Seventeen potential risk factors were evaluated in the meta-analysis.

**Table 1:** Baseline characteristics of all included studies in this meta-analysis

Literature	Publication year	Geographic region	Study accrual period	Study design	Sample size(HC/No HC)	Definition of HC	Risk factors reported	NOS scores
Rostami	2023	Iran	2014.12 − 2021.12	Retrospective	46/154	The presence of sustained painful hematuria with a negative urine culture, in the absence of other explanation such as urinary calculi, gynecologic related bleeding, or general bleeding diathesis.	F1, F8, F15, F16, F17	7
Li	2022	China	2010.01 − 2019.12	Retrospective	26/71	microscopic or macroscopic hematuria and dysuria with a negative bacterial culture in the urine and no other hemorrhagic conditions.	F1, F7, F8, F9, F10, F15, F16	8
Uppugunduri	2017	Canada & Switzerland	2001.01 − 2013.12	Retrospective	11/61	The presence of hematuria (both microscopic and macroscopic) from the initiation of the conditioning regimen up to 30 days post-transplant.	F1, F2, F4, F6, F7, F8, F14	6
Hayden*	2015	USA	2006.12 − 2012.05	Prospective	19/71	Consistent with National Cancer Institute criteria.	F1, F12, F14	7
Cesaro*	2015	Italy	2005.04 − 2011.12	Prospective	20/87	With macrohematuria irrespective of the presence or not of clinical symptoms of dysuria.	F1, F2, F4, F5, F6, F7, F8, F11, F12, F13, F14	8
Laskin*	2013	USA	2010.09 − 2011.12	Prospective	17/71	macroscopic hematuria & macroscopic hematuria with clots & macroscopic hematuria with clots and an elevated creatinine secondary to obstruction.	F1, F2, F4, F5, F6, F8, F9, F10, F11, F12, F13, F14	8
Kloos	2013	Netherlands	2007.01 − 2009.12	Retrospective	14/60	Microscopic or macroscopic hematuria and dysuria with a negative bacterial culture in the urine and no other hemorrhagic conditions.	F1, F2, F8, F9, F10, F11, F12, F13, F15, F17	9
Cheuk	2007	China	1992.05 − 2005.12	Retrospective	14/171	The presence of sustained gross hematuria and symptoms of bladder irritability such as dysuria, frequency or urgency, in the absence of urinary tract infection.	F2, F4, F5, F6, F8	7
Gorczynska	2005	Poland	2001.05 − 2004.12	Retrospective	26/76	The presence of sustained hematuria and symptoms of lower urinary tract irritability, such as dysuria with frequency and urgency.	F1, F2, F8, F12	8
Hale	2003	USA	1992.01 − 1999.12	Retrospective	27/218	Consistent with National Cancer Institute criteria.	F1, F7, F12, F13, F14, F15, F16	7
Cesaro	2003	Italy	1983.06 − 1999.12	Retrospective	44/1174	gross hematuria and thrombocytopenia & gross hematuria and clots & gross hematuria, clots and urethral obstruction.	F1, F2, F3, F4, F5, F6, F11, F12	7
Kondo	1998	Japan	1982.04 − 1996.05	Retrospective	26/240	macroscopic hematuria or sustained (>7 days) microhematuria with clinical signs of cystitis were present.	F1, F2, F3, F11, F12, F13, F14, F15, F16	8

* Ciprofloxacin was used for the prophylaxis of hemorrhagic cystitis

Abbreviation: HC, hemorrhagic cystitis NOS, Newcastle-Ottawa scale

Risk factors: F1, male sex; F2, malignant diseases; F3, allogenic donors; F4, bone marrow transplantation; F5, peripheral blood transplantation; F6, umbilical cord blood transplantation; F7 human leukocyte antigen mismatched; F8, unrelated donor; F9, myeloablative conditioning; F10, reduced intensity conditioning; F11, cyclophosphamide; F12, total body irradiation; F13, busulfan; F14, anti-thymoglobulin; F15, II-IV acute graft-versus-host-disease; F16, chronic graft-versus-host-disease; F17, cytomegalovirus reactivation;

### Incidence and risk factors for HC in this meta-analysis

Among the included studies, the overall incidence rate of HC was 9.9% (276 of 2,764). Male sex, allogeneic donor, human leukocyte antigen (HLA) mismatched donor, unrelated donor, myeloablative conditioning (MAC), busulfan and anti-thymocyte globulin (ATG) use, and cytomegalovirus (CMV) reactivation were risk factors for HC in children undergoing HSCT. Although receiving reduced-intensity conditioning (RIC) was associated with a decreased risk of HC, malignant disease, source of stem cells, use of pre-transplantation cyclophosphamide or total body irradiation (TBI), acute GVHD, and chronic GVHD were not significant risk factors for HC. The pooled results of all potential risk factors are presented in Table 2, and the forest plots of non-significant risk factors are presented in the Supplemental File.

**Table 2:** Pooled analysis of each included risk factor for HC in this meta-analysis

Risk factors	Number of included studies	HC/No HC	Pooled effects			Heterogeneity		Analysis models
			OR	95%CI	*P* value	I^2^, %	*P* value	
Male sex	11	276/2299	1.52	1.16, 2.00	**0.003**	0	0.75	Fixed-effect model
Malignant disease	8	172/1916	1.26	0.83, 1.90	0.28	33	0.16	Fixed-effect model
Allogenic donor	2	70/737	5.28	2.60, 10.74	**<0.00001**	0	0.38	Fixed-effect model
Bone marrow transplantation	5	106/1157	1.10	0.69, 1.76	0.69	0	0.40	Fixed-effect model
Peripheral blood transplantation	4	95/1489	0.56	0.29, 1.11	0.10	0	0.45	Fixed-effect model
Cord blood transplantation	5	106/1053	1.72	0.87, 3.40	0.12	16	0.32	Fixed-effect model
HLA mismatched	3	57/219	1.86	1.00, 3.44	**0.05**	31	0.23	Fixed-effect model
Unrelated donor	9	201/958	1.58	1.10, 2.28	**0.01**	1	0.43	Fixed-effect model
MAC	3	57/202	3.19	1.28, 7.96	**0.01**	0	0.39	Fixed-effect model
RIC	3	57/202	0.31	0.13, 0.78	**0.01**	0	0.39	Fixed-effect model
Pre-transplantation cyclophosphamide	5	121/1622	1.02	0.69, 1.51	0.92	45	0.12	Fixed-effect model
Total body irradiation	8	193/1987	0.94	0.65, 1.36	0.76	0	0.58	Fixed-effect model
Busulfan	5	104/666	2.18	1.33, 3.58	**0.002**	0	0.62	Fixed-effect model
Anti-thymoglobulin	6	120/738	1.65	1.07, 2.54	**0.02**	16	0.31	Fixed-effect model
aGVHD	5	146/663	1.45	0.64, 3.26	0.37	61	0.03	Random-effect model
cGVHD	4	122/603	0.67	0.27, 1.68	0.39	52	0.10	Random-effect model
CMV reactivation	2	60/260	2.65	1.45, 4.84	**0.001**	0	0.73	Fixed-effect model

Abbreviation: HC hemorrhagic cystitis; OR odd ratio; CI confidence interval; HLA human leukocyte antigen; MAC myeloablative conditioning; RIC reduced intensity conditioning; aGVHD acute graft-versus-host-disease; cGVHD chronic graft-versus-host-disease; CMV cytomegalovirus;

### Male sex

**Fig. 2:**
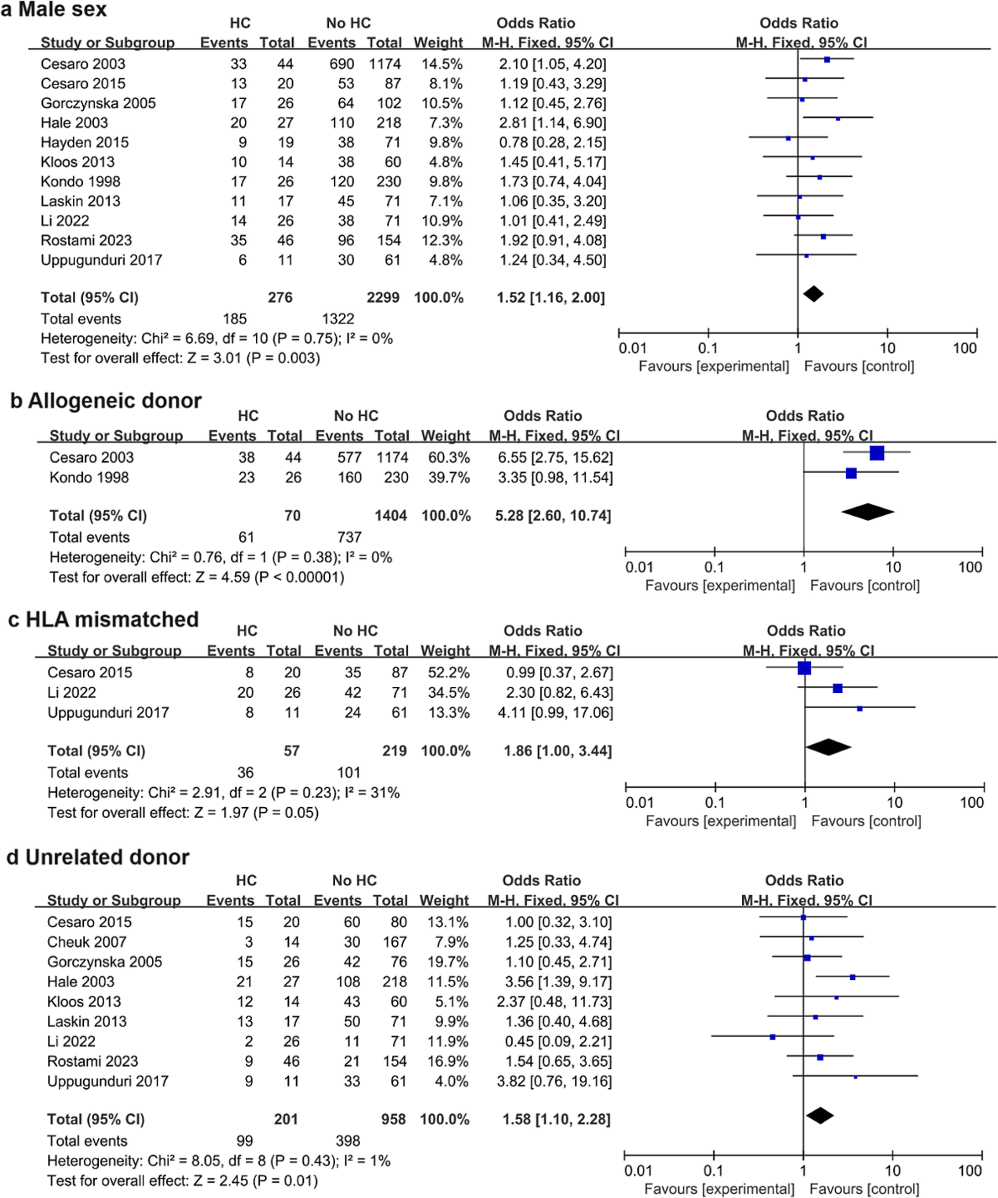
Forest plots of the risk of hemorrhagic cystitis in children undergoing HSCT according to sex (**a**), allogeneic donor (**b**), HLA-mismatched donor (**c**), and unrelated donor (**d**) status

Eleven studies, including 2,575 patients, assessed the association between male sex and the occurrence of HC. The analysis revealed that male patients were at a higher risk of developing HC than female patients (OR = 1.52; 95% CI, 1.16–2.00; *p* = 0.003) (Fig. 2). There was low heterogeneity among the 11 studies (I^2^ = 0, *p* = 0.75), so a fixed-effects model was used to pool the results.

### Malignant disease

Eight studies, including 2,088 patients, were analyzed. The results showed no association between malignant disease and the risk of developing HC in children undergoing HSCT (OR = 1.26; 95% CI, 0.83–1.90; *p* = 0.28) (Figure S2). There was no significant heterogeneity (I^2^ = 33, *p* = 0.16); therefore, a fixed-effects model was used to pool the results.

### Allogenic donor

Two studies, including 807 patients, assessed the association between having an allogenic donor and the risk of developing HC in children undergoing HSCT. Patients with an allogenic donor had a higher risk of developing HC (OR = 5.28; 95% CI, 2.60–10.4; *p* < 0.00001) (Fig. 2). A fixed effects model was used because there was no significant heterogeneity (I^2^ = 0, *p* = 0.38).

### Sources of stem cell

The pooled results indicated that there was no association between the source of bone marrow transplantation and HC in children undergoing HSCT, with no heterogeneity (OR = 1.10; 95% CI, 0.69–1.76; *p* = 0.69; I^2^ = 0, *n* = 1263) (Figure S3). Similarly, there were no statistically significant differences between peripheral blood transplantation (OR = 0.56; 95% CI, 0.29–1.11; *p* = 0.10; I^2^ = 0, *n* = 1584) (Figure S4) or cord blood transplantation (OR = 1.72; 95% CI, 0.87–3.40; *p* = 0.12; I^2^ = 16, *n* = 1159) (Figure S5) and the risk of developing HC in children undergoing HSCT, with no heterogeneity.

### HLA-mismatched donor

Analysis of three studies including 276 patients showed that in children undergoing HSCT, patients with an HLA-mismatched donor had a higher risk of developing HC than those with matched donors (OR = 1.86; 95% CI, 1.00–3.44; *p* = 0.05) (Fig. 2). A fixed-effects model was used, as there was no significant heterogeneity (I^2^ = 31, *p* = 0.23) among the studies.

### Unrelated donor

Nine studies, including 1,159 patients, were analyzed. The analysis revealed that in children undergoing HSCT, patients who received cells from an unrelated donor had a higher risk of developing HC (OR = 1.58; 95% CI, 1.10–2.28; *p* = 0.01) (Fig. 2). A fixed effects model was used because there was no significant heterogeneity (I^2^ = 1, *p* = 0.43).

### Conditioning regimen

**Fig. 3:**
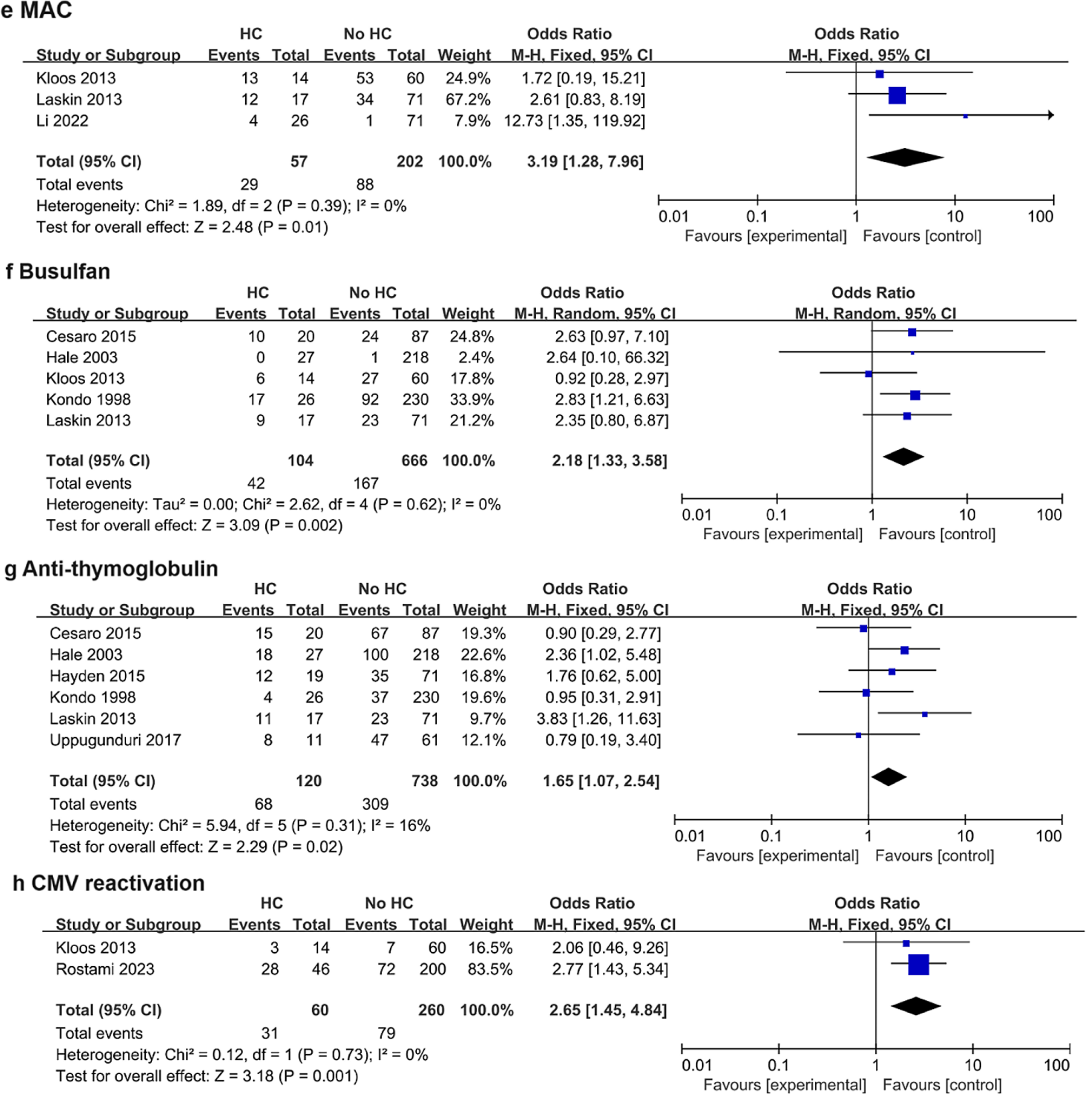
Forest plots of the risk of hemorrhagic cystitis in children undergoing HSCT according to myeloablative conditioning (MAC) (**e**), busulfan (**f**), anti-thymoglobulin (**g**), and cytomegalovirus (CMV) reactivation (**h**) status

Regarding the conditioning regimen, MAC was associated with an increased risk of developing HC in children undergoing HSCT (OR = 3.19; 95% CI, 1.28–7.96; *p* = 0.01) (Fig. 3), whereas receiving RIC was associated with a reduced risk of developing HC (OR = 0.31; 95% CI, 0.13–0.78; *p* = 0.01) (Figure S6), with no heterogeneity (I^2^ = 0, *n* = 259).

### Conditioning agents in HSCT

Regarding the agents used in the conditioning regimen, the use of busulfan (OR = 2.18; 95% CI, 1.33–3.58; *p* = 0.002) (Fig. 3) or ATG (OR = 1.65; 95% CI, 1.07–2.54; *p* = 0.02) (Fig. 3) was associated with an increased risk of developing HC in children undergoing HSCT, with no heterogeneity (busulfan: I^2^ = 0, *n* = 770; and ATG: I^2^ = 16, *n* = 858). In contrast, the use of pre-transplantation cyclophosphamide (OR = 1.02; 95% CI, 0.69–1.51; *p* = 0.92) (Figure S7) or TBI (OR = 0.94; 95% CI, 0.65–1.36; *p* = 0.76) (Figure S8) was not associated with the risk of developing HC in children undergoing HSCT, with no heterogeneity (cyclophosphamide: I^2^ = 45, *n* = 1843; and TBI: I^2^ = 0, *n* = 2180).

### Acute and chronic GVHD

Neither acute GVHD (OR = 1.45; 95% CI, 0.64–3.26; *p* = 0.37) (Figure S9) nor chronic GVHD (OR = 0.67; 95% CI, 0.27–1.68; *p* = 0.39) (Figure S10) was associated with an increased risk of developing HC in children undergoing HSCT. However, there was significant heterogeneity among the included studies (acute GVHD: I^2^ = 61, *n* = 809; and chronic GVHD: I^2^ = 61, *n* = 725); therefore, random effects models were used to pool the results.

### CMV reactivation

Only two studies, including 320 patients, investigated the relationship between CMV reactivation and HC development. Patients with CMV reactivation had an increased risk of HC after undergoing HSCT (OR = 2.65; 95% CI, 1.45–4.84; *p* = 0.001) (Fig. 3). A fixed-effects model was used, as there was no significant heterogeneity (I^2^ = 0, *p* = 0.73).

## Discussion

This is the first meta-analysis to explore the risk factors for HC in children undergoing HSCT. Zhou et al. [22] reported the risk factors related to BK virus-associated HC (BKV-HC). They found that male sex, haploidentical donor, MAC, acute GVHD, chronic GVHD, and CMV reactivation were risk factors for BKV-HC in patients who underwent allogeneic HSCT. Owing to the limited number of included studies, they did not perform a subgroup analysis of pediatric patients and only investigated virus-induced HC. Therefore, the risk factors for pediatric patients undergoing HSCT remain unclear. This meta-analysis included 12 studies involving 2,764 patients that explored the risk factors for HC in children undergoing HSCT, and 17 potential risk factors were evaluated. The results of the pooled analysis indicated that male sex, allogeneic donor, HLA-mismatched donor, unrelated donor, MAC, use of busulfan or ATG, and CMV reactivation were risk factors for HC in children undergoing HSCT.

Our study revealed that boys were more likely to develop HC after HSCT than girls, which is consistent with the results of previous studies [9, 23, 24]. A possible reason for this finding may be that estrogen exerts a protective effect in the bladder mucosa, which contributes to a lower incidence of HC in female HSCT recipients than in male recipients [25].

In our analysis, no significant differences were observed in the risk of HC between patients with malignant and nonmalignant diseases. All studies included in this meta-analysis consistently indicated that patients with malignant disease did not exhibit a higher risk of HC than those with nonmalignant disease. However, in patients with malignant disease, intensive chemotherapy may cause injury to the bladder mucosa and contribute to the occurrence of HC after HSCT. Although our analysis did not suggest that malignant disease is a risk factor for HC in children undergoing HSCT, further studies are warranted to explore this relationship.

The types of HSCT include autologous and allogeneic, owing to the different sources of stem cells (the patients or others). Recipients of allogeneic transplantation may have a higher incidence of HC [14, 26], which is consistent with our results. We speculate that this association may be attributable to the higher dose of cyclophosphamide (which can injure the bladder mucosa) employed in the conditioning regimen of allogeneic transplant recipients. However, the reliability of this finding is constrained due to the inclusion of only two studies. Further studies with large sample sizes are warranted to elucidate the relationship between HC and the type of HSCT.

The stem cell sources generally included bone marrow, peripheral blood, and cord blood. The results revealed that the risk of HC did not differ significantly according to the source of stem cells, which is consistent with the findings of previous studies [10, 18, 27]. Although our analysis did not find an association between cord blood transplantation and the risk of HC in pediatric HSCT recipients, Jiang et al. [28] reported that HLA matching ≤ 6/8 was an independent risk factor for the occurrence of late-onset HC. HLA compatibility of cord blood units may play an essential role in the relationship between cord blood transplantation and the occurrence of HC. However, we could not assess this relationship owing to the limited data available. Further studies are required to assess the relationship between HLA mismatching and the risk of HC.

Previous studies have reported that the risk of HC is higher in HSCT recipients who receive cells from an unrelated donor [19, 29], which is consistent with the findings of our study. However, other studies have found that an HLA-mismatched donor is not a risk factor for HC [5, 15]. Our analysis results indicated that receiving cells from both HLA-mismatched and unrelated donors was associated with an increased risk of HC. This may be because recipients of cells from an unrelated donor require more intensive immunosuppression therapy, which may contribute to the development of HC [2]. Therefore, a matched-related donor is preferred to prevent the occurrence of HC in children undergoing HSCT. However, this conclusion may be unreliable owing to the limited number of studies included, and further high-quality studies are warranted to further explore the relationship between HLA-mismatched donors and HCs.

According to the different intensities of immunosuppression, the conditioning regimen was divided into two general categories: MAC and RIC. The HC risk was higher in patients who received MAC than in those who received RIC, which is consistent with the findings of several previous studies [5, 30]. This finding can be attributed to the lower toxicity of RIC, which causes milder damage to the mucosa of the bladder and, subsequently, a lower incidence of HC. Cyclophosphamide administration has previously been reported to be associated with an increased risk of HC [31]; however, our results indicate that it does not increase the risk of HC [31]. This may be because most of the studies included in this analysis investigated late-onset HC, whereas cyclophosphamide is more often associated with early-onset HC [32]. Busulfan, an alkylating agent commonly used in conditioning regimens, has previously been reported to be associated with an increased risk of HC [6], which is consistent with the results of this meta-analysis. The increased risk of HC associated with busulfan use may be attributable to the urothelial changes caused by busulfan [33]. The use of ATG was also associated with an increased risk of HC in this meta-analysis. This may be attributable to the higher risk of viral infections in patients who receive this drug [34]. Similarly, Fu et al. [35] suggested that a higher dose of ATG may be associated with an increased risk of HC in haploidentical HSCT recipients. TBI is another key component of conditioning for HSCT. Several previous studies have not found an association between TBI and the risk of HC [12, 18, 27], which is consistent with the results of our analysis.

GVHD is another common complication in patients undergoing HSCT, which limits the use of HSCT and is a leading cause of morbidity and mortality [36]. Acute GVHD has been reported to be associated with an increased risk of HC [5]. This may be attributable to the immunosuppressive therapy used for treating acute GVHD, which increases the risk of viral infection and subsequently causes the occurrence of HC. Alternatively, HC may be a manifestation of GVHD, which generally involves multiple systems [37]. However, the pooled results of our meta-analysis indicate that acute GVHD does not increase the risk of HC. However, this finding may be unreliable owing to the limited number of included studies and the significant heterogeneity between studies. Several previous studies have explored the relationship between chronic GVHD and HC but have found no association [9, 19], which is consistent with our results. However, this finding may be unreliable owing to the limited data available in the included studies and the relatively high heterogeneity between studies.

Our analysis indicated that CMV reactivation was a risk factor for HC in HSCT recipients. Zhang et al. [38] reported that the incidence rate of HC was higher in patients with CMV infection than in those without CMV infection. This may be because CMV infection causes impaired T-cell immunity and weak T-cell responses, which contribute to an increased risk of BKV infection and, consequently, the occurrence of HC [39]. However, this finding may be unreliable owing to the small number of studies included in the analysis. Further studies with large sample sizes are warranted to further explore the relationship between CMV reactivation and HC.

Owing to the limited data available in the included studies, we could not analyze the risk factors for the severity of HC. However, Leung et al. [40] reported that a 3-log increase in the BKV load in adult patients undergoing HSCT was an independent risk factor for severe HC. Another recently published study showed that BKV infection and multiple infections were predictors of HC severity in children undergoing HSCT [41]. Ost et al. [37] reported that the severity of acute GVHD was related to the severity of HC. BKV infection and GVHD may play key roles in the development of HC and cause more severe disease. Therefore, managing BKV infection and GVHD is important for preventing HC in children undergoing HSCT.

Other potential risk factors reported in previous studies include age and T-cell depletion. However, owing to the limited data available and the lack of standardization of reporting, these factors were not investigated in this meta-analysis. Previous studies have reported that in pediatric HSCT recipients, the incidence of HC is lower in younger children [6, 11, 42]. This may be attributable to the lower incidence of viral infection in younger children. Regarding T-cell depletion, a previous study found that patients with T-cell depletion had a higher risk of developing HC [9]. This may be because T-cell depletion results in substantial immunosuppression, which increases the risk of viral infection and HC. Further high-quality prospective studies are warranted to further explore the relationship between these factors and the development of HC.

Therapeutic options for HC are diverse and differ according to the disease severity and the presence of viral infection. Preventive therapy and conservative and supportive therapy should be used as the first steps in non-severe cases. In patients with more severe HC, continuous bladder irrigation and intravesical therapy may be considered. Moreover, in very severe cases, surgical procedures may be required to remove clots and stop bleeding [8]. Cidofovir, an antiviral drug, has high specificity against the BKV and has been shown to be effective in preventing and treating BKV-HC [43, 44]. Other antiviral drugs, such as leflunomide, have also been reported to be effective against BKV-HC [45, 46]. Virus-specific T cells (VSTs), which are derived from a patient’s stem cell donor (donor-derived) or a third-party donor, are a safe and effective therapy for viral infections in children undergoing HSCT [47, 48]. In a cohort of 56 patients undergoing HSCT, gross hematuria was completely resolved in 13 of 14 patients with BKV-HC who received VST infusion for six weeks, whereas four of the five patients who did not receive VST infusion developed disease progression [49]. Another clinical trial conducted by Olson et al. [50] reported that the clinical response of patients with HC who received VST was better than that of historical controls. These findings indicate that VSTs may be a feasible option for treating HC, especially BKV-HC, in patients undergoing HSCT. Other treatment strategies, such as estrogen, hyperbaric oxygen therapy, and mesenchymal stromal cells, have also been reported to be effective and can be used as alternative strategies.

This meta-analysis has several limitations. First, combining prospective and retrospective studies may result in potential bias. Second, owing to the limited number of included studies, it was not possible to perform multivariate or subgroup analyses. Third, the large time span of included studies and different transplantation protocols may limit the generalizability of our findings. Fourth, the criteria for the diagnosis of HC differed in different studies, which may contribute to bias.

## Conclusions

Our meta-analysis suggested that male sex, allogeneic donor, HLA-mismatched donor, unrelated donor, MAC, use of busulfan or ATG, and CMV reactivation were potential risk factors for HC in pediatric HSCT recipients. However, additional prospective studies with large sample sizes are required to further explore the risk factors for HC in children undergoing HSCT.

### Electronic supplementary material

Below is the link to the electronic supplementary material.

Supplementary Material 1

## Data Availability

All data generated or analyzed during this study are included in this published article [and its supplementary information files].

## Abbreviations

ATG: Anti-thymocyte globulin
BKV: BK virus
CI: Confidence interval
CMV: Cytomegalovirus
GVHD: Graft versus host disease
HC: Hemorrhagic cystitis
HLA: Human leucocyte antigen
HSCT: Hematopoietic stem cell transplantation
MAC: Myeloablative conditioning
NOS: Newcastle Ottawa Scale
OR: Odds ratio
RIC: Reduced-intensity conditioning
VST: Virus-specific T-cells
