# Supplementary material for: Risk factors for hemorrhagic cystitis in children undergoing hematopoietic stem cell transplantation: a systematic review and meta-analysis
Source: BMC Pediatr. 2024 May 14;24:333. doi: 10.1186/s12887-024-04815-x (PMC11092211; doi:10.1186/s12887-024-04815-x)
Supplement: Supplementary file 1 — Supplementary Material 1 [file 12887_2024_4815_MOESM1_ESM.docx]

**Risk factors for** **hemorrhagic cystitis in children undergoing** **hematopoietic stem cell transplantation: A systematic review and meta-analysis**

***ONLINE SUPPLEMENT***

**Supplemental Figure 1:** A funnel plot of studies included in male sex analysis.

**Supplemental Figure 2-10:** Forest plots of non-significant risk factors.

**Supplemental Table 1:** Quality assessment of studies included in this meta-analysis.

**Search strategies in PubMed:** ((("Cystitis"[Mesh]) OR ((Cystitis[Title/Abstract]) OR (Cystitides[Title/Abstract]))) AND (((Hematopoietic Stem Cell Transplantation[Title/Abstract]) OR (Stem Cell Transplantation, Hematopoietic[Title/Abstract])) OR (Transplantation, Hematopoietic Stem Cell[Title/Abstract]))) AND ((((((((Infant[Title/Abstract]) OR (Infants[Title/Abstract])) OR (Child[Title/Abstract])) OR (Children[Title/Abstract])) OR (Pediatrics[Title/Abstract])) OR (Pediatric[Title/Abstract])) OR (Adolescent[Title/Abstract])) OR (Adolescents[Title/Abstract]))

**Supplemental Figure 1**: A funnel plot of studies included in male sex analysis.


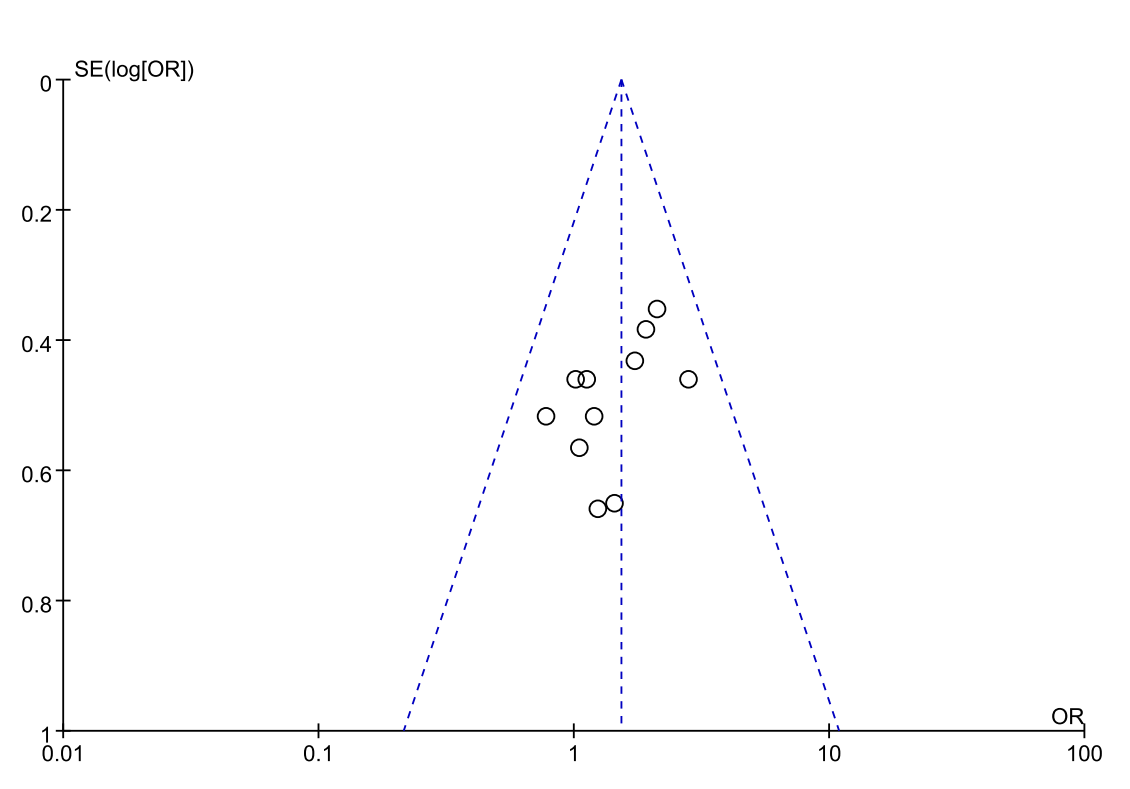


**Supplemental Figure 2:** Forest plot of malignant disease.


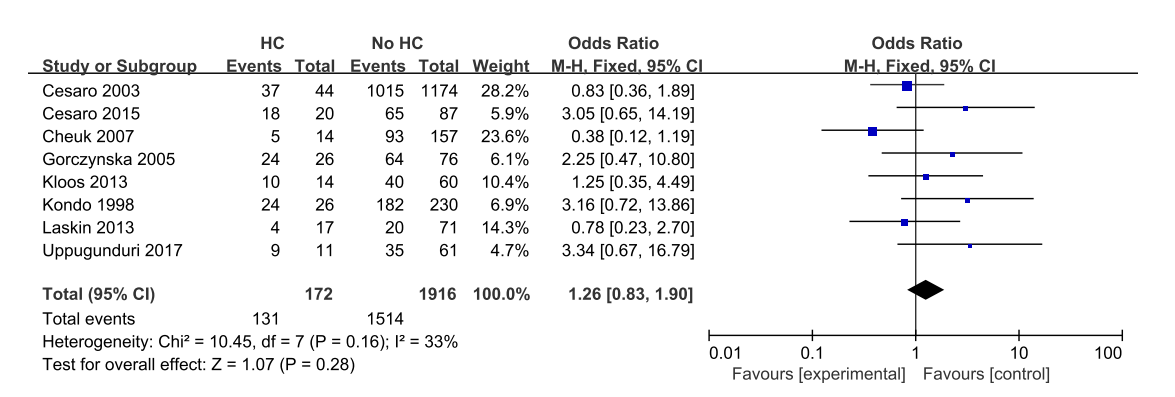


**Supplemental Figure 3:** Forest plot of bone marrow transplantation.


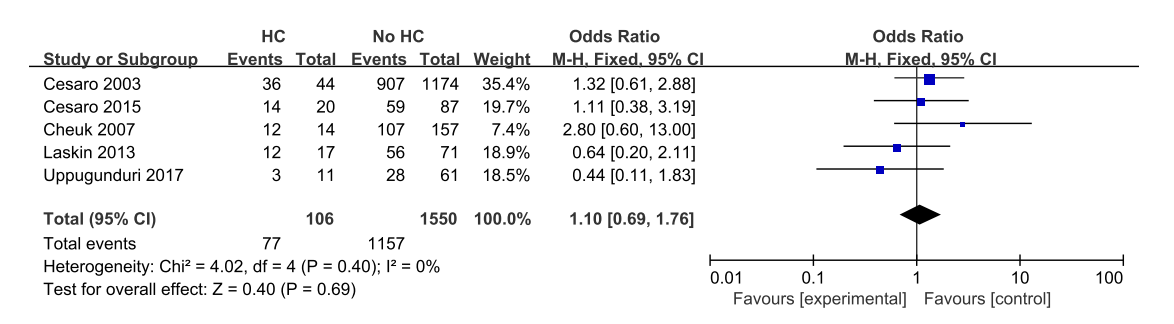


**Supplemental Figure 4:** Forest plot of peripheral blood transplantation.


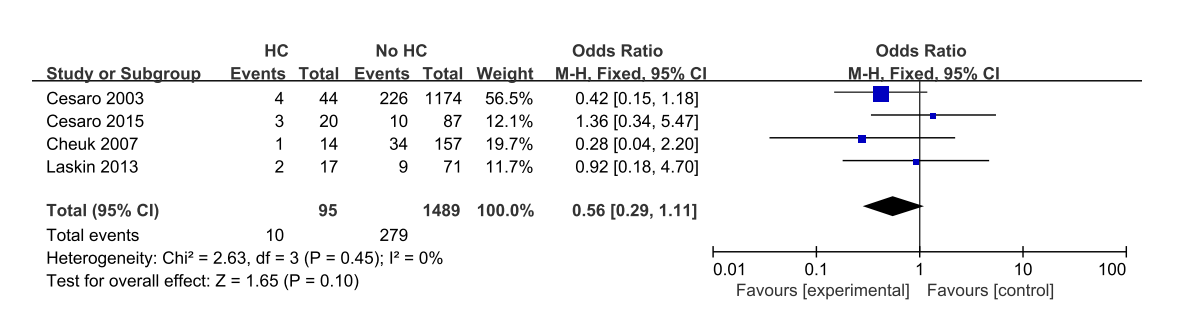


**Supplemental Figure 5:** Forest plot of cord blood transplantation.


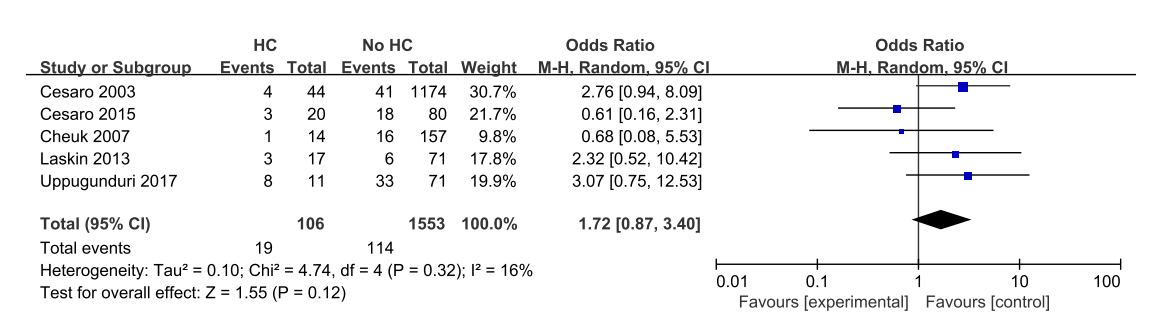


**Supplemental Figure 6:** Forest plot of reduced intensity conditioning.


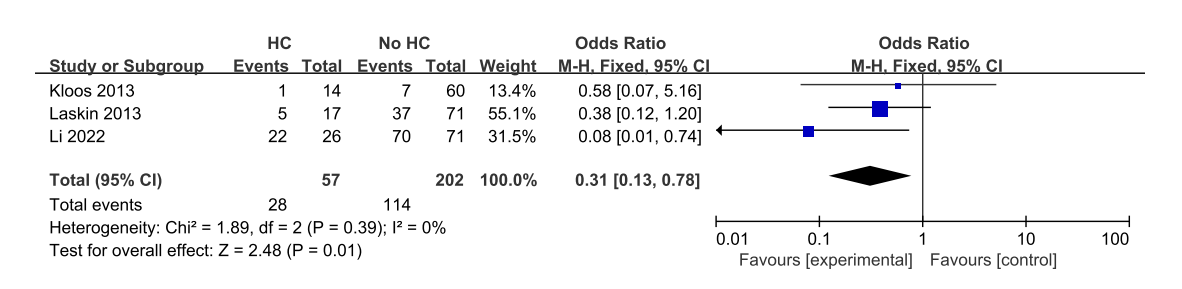


**Supplemental Figure 7:** Forest plot of pre-transplantation cyclophosphamide.


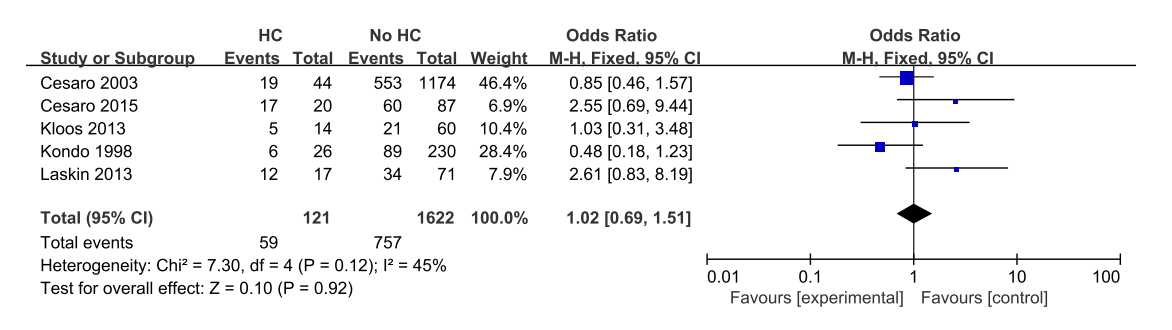


**Supplemental Figure 8:** Forest plot of total body irradiation.

**
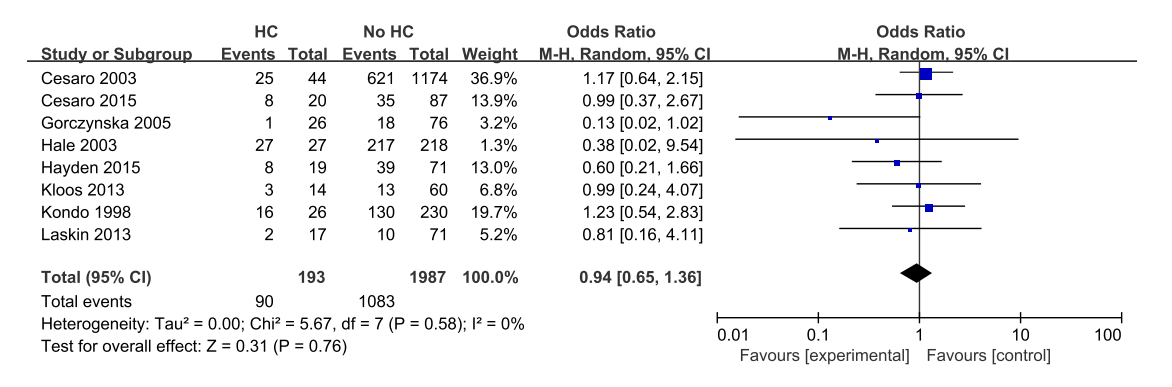
**

**Supplemental Figure 9:** Forest plot of acute graft-versus-host disease.

**
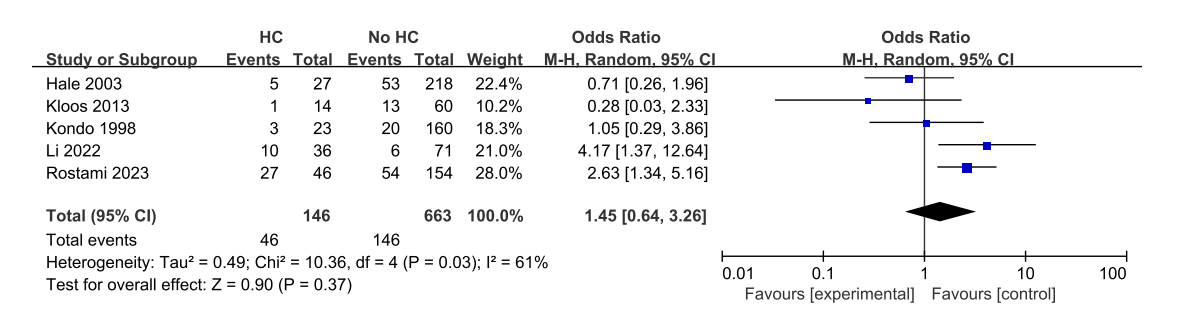
**

**Supplemental Figure 10:** Forest plot of chronic graft-versus-host disease.


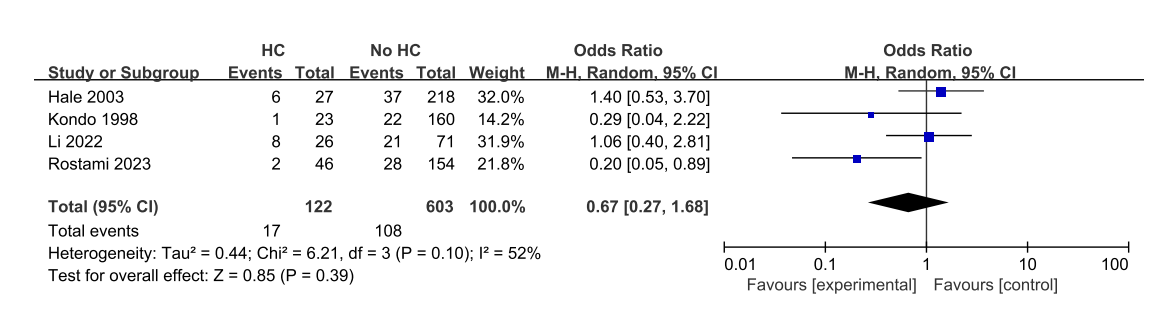


**Supplemental Table 1:** Quality assessment of studies included in this meta-analysis.

| Study | Selection | | | Comparability | | | Outcome/Exposure | | | Scores |
| --- | --- | --- | --- | --- | --- | --- | --- | --- | --- | --- |
|  | Representativeness of the exposed cohort | Selection of the non-exposed cohort | Determination of exposure | Ascertainment of no outcome before the study | Matching the most important factor | Control of confounding factors | Evaluation of outcome | Adequacy of follow up | Completeness of follow up |  |
| Rostami | 1 | 1 | 0 | 1 | 1 | 1 | 1 | 0 | 1 | 7 |
| Li | 1 | 1 | 1 | 0 | 1 | 1 | 1 | 1 | 1 | 8 |
| Uppugunduri | 0 | 1 | 1 | 1 | 0 | 0 | 1 | 1 | 1 | 6 |
| Hayden | 1 | 0 | 1 | 1 | 0 | 1 | 1 | 1 | 1 | 7 |
| Cesaro | 1 | 1 | 1 | 1 | 1 | 1 | 0 | 1 | 1 | 8 |
| Laskin | 1 | 1 | 0 | 1 | 1 | 1 | 1 | 1 | 1 | 8 |
| Kloos | 1 | 1 | 1 | 1 | 1 | 1 | 1 | 1 | 1 | 9 |
| Cheuk | 1 | 1 | 0 | 1 | 1 | 0 | 1 | 1 | 1 | 7 |
| Gorczynska | 1 | 1 | 1 | 0 | 1 | 1 | 1 | 1 | 1 | 8 |
| Hale | 1 | 1 | 0 | 1 | 1 | 1 | 0 | 1 | 1 | 7 |
| Cesaro | 1 | 0 | 1 | 1 | 0 | 1 | 1 | 1 | 1 | 7 |
| Kondo | 1 | 1 | 1 | 0 | 1 | 1 | 1 | 1 | 1 | 8 |
